# How do changes in motivation to prevent pregnancy influence contraceptive continuation? Results from a longitudinal study with women who receive family planning services from Community Pharmacists and Patent and Proprietary Medicine Vendors in Nigeria

**DOI:** 10.1186/s12978-022-01326-9

**Published:** 2022-02-08

**Authors:** Sara Chace Dwyer, Sikiru Baruwa, Emeka Okafor, Babajide Oluseyi Daini, Osimhen Ubuane, Aparna Jain

**Affiliations:** 1 Population Council, 4301 Connecticut Ave NW # 280, Washington, DC 20008 USA; 2 Population Council, Abuja, Nigeria; 3 Society for Family Health, Abuja, Nigeria

**Keywords:** Family planning, Pregnancy motivations, Drug shops, Pharmacies, Task sharing, Private sector, Nigeria

## Abstract

**Background:**

Studies have shown that motivation to avoid pregnancy is associated with contraceptive use and continuation. These motivations can change, however, even within a short period of time. This paper uses longitudinal data to look at women’s motivation to avoid pregnancy at two time points, and how changes in motivation influence contraceptive continuation.

**Methods:**

Data for this analysis came from an evaluation of the IntegratE project which seeks to expand access to family planning (FP) in Nigeria through community pharmacies and drug shops. 491 women were interviewed within 10 days after receiving a FP service from these sources and again approximately 9 months later. The dependent variable was contraceptive continuation at the follow-up interview. A categorical independent variable was used to represent changes in motivation to avoid pregnancy from enrollment to the follow-up interview. Univariate and multivariate logistic regression models were used to assess the association between changes in motivation and contraceptive continuation.

**Results:**

89% of women continued using contraception approximately 9 months after the enrollment interview. Women who remained highly motivated to avoid pregnancy were significantly more likely to continue using contraception compared to women who became more motivated (AOR 2.5; 95% CI 1.0–6.0). Women who became less motivated were 64% less likely to continue using contraception compared to who became more motivated (AOR 0.36 95% CI 0.1–0.9).

**Conclusion:**

FP providers, including private sector pharmacists and drug shop owners, should continuously check-in with women about their motivations around pregnancy to support continuation among those who wish to avoid pregnancy.

## Plain language summary

Previous research has found women’s motivations to avoid pregnancy- how important it is to avoid pregnancy now- is associated with their contraception use. Yet women’s motivations to avoid pregnancy often change, even within a short period of time. This paper looks at whether changes in motivation to avoid pregnancy are associated with continued use of contraception in Nigeria.

We used data from a larger evaluation of the IntegratE project which seeks to expand access to family planning (FP) in Nigeria through community pharmacies and drug shops. 491 women were interviewed within 10 days after receiving a FP service (counseling, referral or FP method) from these sources and again 9 months later. We then used logistic regression models to assess whether changes in motivation to avoid pregnancy between receiving a FP service to 9 months later is associated with continued use of FP.

We found that many women (89%) continued using contraception approximately 9 months after the enrollment interview. Women who remained highly motivated (reported it was very important to avoid pregnancy at both interviews) were more likely to continue using contraception compared to women who became more motivated between the two interviews. Women who became less motivated were 64% less likely to continue using contraception compared to who became more motivated. Our results suggest that FP providers, including private sector pharmacists and drug shop owners, should continuously check in with women about their motivations around pregnancy to support continuation contraceptive use among those who wish to avoid pregnancy.

## Background

Contraceptive discontinuation contributes to approximately one-third of unmet need globally [1]. Given that discontinuation contributes to a sizable portion of unmet need, numerous studies have explored reasons associated with discontinuation among women who wish to avoid pregnancy. Method-related factors associated with discontinuation include experience of side effects, women’s disatisfaction with the method, and the type of method selected (for example, short-acting versus long-acting or hormonal versus nonhormonal methods) [2–10].

Studies have also identified non-method related factors associated with contraceptive continuation. These factors include experiencing an unintended pregnancy, having partner’s support, being older in age, achieving a desired number of children, higher number of living children, higher number of male children, being in school or working, and discussing FP with a friend [6, 7, 9, 11–13]. Fertility intentions (the desire for a certain number of children and the intended timing of a first birth and subsequent spacing between births [14]) have also been shown to be associated with contraceptive use [15] and continuation [12, 13]. Yet women’s fertility intentions can and often change, even within a short period of time [15–17]. For example, one study in the US has found that while 39% of women who were interviewed three times within a year reported being uncertain if they wanted more children, only 9% consistently reported uncertainty [16]. Similarly, a longitudinal study in India has found that among women who reported wanting a child in two or more years, only 5% of those women consistently reported that preference during subsequent interviews [17].

Asking a woman about her fertility intentions focuses on if and when (intentions for the future) a woman would like to become pregnant. Recently, researchers have been exploring the relationship between women’s present motivations to avoid pregnancy and their contraceptive use. Studies have shown that a woman’s motivation to avoid pregnancy and intent to use contraception are associated with method selection [11] and contraceptive continuation [7, 18]. A recent study in India has found that both cognitive (i.e., how important is it to avoid pregnancy) and affective (i.e., degree of happiness if you found out you were pregnant) attitudes are significantly associated with contraceptive continuation 9 months after method adoption [18]. The same study has found that many women often have conflicting cognitive and affective attitudes towards pregnancy [18], consistent with studies in the United States [19, 20]. These studies in the United States also have found that women who hold ambivalent or conflicting views towards pregnancy are more likely to not use contraception at all [19] or use contraception inconsistently [20]. The study in India, however, has found no difference in contraceptive continuation between women who were ambivalent towards pregnancy and those who held consistent cognitive and affective attitudes towards pregnancy [18]. A study in Malawi has found that women who were indifferent to being pregnant were more likely to use contraceptive than those who held a strong desire to avoid pregnancy [21]. A study among teens in the US has found young women who had low motivations to avoid pregnancy used contraception inconsistently [22].

### Expanding access to family planning through private sector pharmacies and drug shops

In Nigeria, about 15% of all women of reproductive age (15–49) have an unmet need for family planning (FP)- 10% for spacing and 5% for limiting pregnancies [23]. About 41% of women who begin using a contraceptive method discontinue that method within 12-months and the main reasons cited for discontinuation include desire to become pregnant, side effects/health concerns and infrequent sex [23]. As part of the Nigerian Federal Ministry of Health’s strategy to expand access to FP, they are exploring task sharing certain FP services to private sector Community Pharmacists (CPs) and Patent and Proprietary Medicine Vendors (PPMVs). Task sharing involves delegating specific tasks among healthcare teams and often from high-skilled healthcare workers to those with fewer qualifications, where appropriate [24]. Task sharing FP within public sector is common [25, 26] but task sharing to private sector pharmacies and drug shops, however, is limited despite being identified a promising high impact practice to expand access to FP [27].

CPs and PPMVs are important sources for primary health care for many Nigerians [28, 29]. They are also popular sources of FP although that they are not currently recognized as formal FP providers. For example, 22% of modern contraceptive users’ report receiving their last method from a PPMV [23]. PPMVs are an especially popular source of care as they are widespread through rural and urban parts of Nigeria [29]. PPMVs are not required to receive a standard training or degree for licensure and are only authorized to provide over the counter medications [29, 30]. CPs are fewer in number compared to PPMVs and are similar to small pharmacies in other settings. CPs are required to complete a 5-year Pharmacy degree before receiving a license to own and operate a community pharmacy. Previous studies have shown that while many PPMVs provide FP services, that they do not have the required knowledge to do so [31, 32]. PPMVs’ knowledge to provide injectable contraceptives has been shown to increase with training [33, 34] and PPMVs’ FP clients are also generally satisfied with the services received [31, 33].

This paper uses longitudinal data from Kaduna and Lagos states, Nigeria to assess changes in motivations to avoid pregnancy measured at two time-points approximately 9 months apart among women who received FP services from CPs and PPMVs. Specifically, the paper looks at whether changes in their motivation to avoid pregnancy influences contraceptive continuation.

### The IntegratE project

IntegratE is a 4-year project (2017–2021) that collaborates with the Federal Ministry of Health to pilot a three-tiered accreditation system for PPMVs. Under the accreditation systems, PPMVs are stratified into three tiers based on their healthcare qualifications. Those without healthcare qualifications are categorized as Tier 1, those with a background in Nursing and Midwifery, Community Health Extension, or were one a Community Health Officer are categorized as Tier 2, and those with pharmacy technician’s certificate are categorized as Tier 3. Under the pilot accreditation system, Tier 1 PPMVs participate in a 3-day training on FP counseling, provision of condoms, cycle beads and oral contraceptive refills, referrals for FP methods, and documenting FP services. Tier 2 and Tier 3 PPMVs receive the same training as Tier 1 PPMVs plus an additional 3-day training on injectable administration, and implant insertion and removal. As CPs must obtain a pharmacy degree for licensure, they function outside of the pilot accreditation system but receive the same training as Tier 2 and Tier 3 PPMVs.

Between July 2018 and September 2019, 894 CPs and PPMVs enrolled in the IntegratE project were trained in FP based on their tier. The FP counseling, referral and FP reporting sessions were classroom-based. CPs, Tier 2 and Tier 3 PPMVs were required to competently complete 12 implant insertions and 3 removals as part of their clinical training in nearby public health facilities. The IntegratE project and state teams (Pharmacy Council of Nigeria, National Association of Patent and Proprietary Medicine Dealers, State Ministry of Health and State Primary Health Care Development Agency) provided supportive supervision approximately 3 months after the training.

### Study sites

As of 2020, the IntegratE project has been implemented in Lagos and Kaduna states. Lagos state has a population of 9,013,534 and is located in the southern part of Nigeria. Kaduna state has a population of 6,113,503 and is in northern part of the country [36]. According to the 2018 Nigeria Demographic and Health Survey, the total fertility rate is relatively high in Kaduna state (5.9 births per woman) compared to Lagos state (3.4 births per woman). The modern contraceptive prevalence rate among married women of reproductive age varies across the two states from 29% in Lagos to 14% percent in Kaduna [23]. The contraceptive prevalence rate for all methods is 59% and 15% in Lagos and Kaduna, respectively [23].

## Methods

### Data source

We used longitudinal data from an on-going evaluation of the IntegratE project (2018–2021) for this analysis. In 2019, trained research assistants interviewed women who received FP services (counseling, referral, condoms, and/or oral, injectable and implant contraceptives) from an IntegratE-trained CP or PPMV within 10 days of receiving that FP service. IntegratE CPs and PPMVs provided female FP clients between the ages of 16–49 with general information about the study and requested their permission to share their contact details with the research team. Research assistants trained on research ethics, the study’s design, informed consent, and conducting telephone interviews, then called 837 clients to confirm their eligibility. Eligible respondents were between 16 and 49 (women 16–17 had to be married to be considered an emancipated minor), received a FP service from a IntegratE-trained CP or PPMV, and owned their own cell phone. Phone ownership was used as an eligibility criterion to facilitate re-interviewing women across the two states while also ensuring their privacy would not be compromised if they shared a phone with another household member. Respondents could be continuing FP users, method switchers, or new to FP altogether. If eligible, the research assistants provided potential respondents with details of the study including the objectives, what was expected of them as a respondent, and potential risks and benefits to participating. Those who agreed were interviewed over the phone and compensated 500 Naira (Approximately 1.39 USD) in phone credit. Research assistants also requested to re-contact participants in approximately 9 months. The study protocol received IRB approvals from the Population Council (Protocol 878) and National Health Research and Ethics Committee.

Research assistants used tablets to administer a quantitative client enrollment interview that included questions on socio-demographic characteristics, current and previous contraceptive use, the quality of care received, motivations to avoid pregnancy, and general perceptions of their experiences receiving services from the CP or PPMV. The same respondents were contacted by phone approximately 9–11 months later for the follow-up interview and were asked questions related to their current contraceptive use, motivations to avoid pregnancy, experience of side effects, continued use of FP services from CPs and PPMVs, and COVID-19. Of the 837 women contacted, a total of 596 women were reached, gave informed consent and were interviewed at enrollment. A total of 517 were interviewed at follow-up (13% lost to follow-up). We conducted Pearson chi2 tests to compare respondent characteristics of women interviewed at both time points against those included those who were loss-to-follow-up. There were no significant differences between the two samples in the characteristics reported (Annex 1). The sample size formula was based on study that assessed the quality of care of FP services from PPMVs [33].

### Dependent variable

The dependent variable is contraceptive continuation at the follow-up interview. At enrollment, all women received a FP method, or a FP referral, from the CP or PPMV that they saw. At follow-up, interviewers asked respondents if they were currently using the same, different, or no method to avoid or delay pregnancy. We dichotomized the dependent variable where FP continuers were coded as 1 and included respondents who reported using the same FP method that they received at enrollment or a different method. Respondents who stopped using FP altogether were considered discontinuers and coded as 0.

### Independent variable

The main independent variable is motivation to avoid pregnancy and was adapted from a similar study conducted in India [17, 18] and in the US [22]. Women were asked “*How important is it for you to avoid pregnancy now*” at both interviews. Response options were on a 4-point Likert scale from very important, important, not important, and not important at all. A categorical variable was created combining responses at both time points using the following 4 categories: 1 = remained highly motivated (reported very important at both interviews); 2 = increase in motivation (importance increased from enrollment to follow-up); 3 = decrease in motivation (importance decreased from enrollment to follow-up); 4 = remained either motivated not motivated (reported important at both interviews or not/at all important at both time points). Response of not important/not at all important at both interviews resulted in a small sample and was therefore combined with remained motivated at both interviews.

Additional covariates explored and included in the multivariate model include age, education, marital status, number of living children, employment status, past contraceptive use, experience of side effects as reported at follow-up, and state of residence. For experience of side effects, we created a categorical variable from three questions included in the follow-up interview. Women who said they were using the pill, injectable and implant were asked if they were currently experiencing side effects. If they responded No, they were then asked if they had experience side effects since the enrollment interview. Those who responded Yes to currently experiencing side effects or Yes to having had experienced side effects since enrollment were coded as 1 and those who said No to both questions were coded as 0. Those who did not respond or were skipped from this question because they were using another method (for example, cycle beads or condoms) were coded as 2 No Response. All women who discontinued their method were also asked whether they experience side effects as a result of their method. Those who said Yes were coded as 1, those who said No were coded as 0 and anyone who did not responded were coded as 2.

Ten observations were missing from the education variable and therefore coded as 0, did not report receiving secondary education or higher. Additional covariates including type of method selected and type of provider seen were explored but not statistically significant and therefore excluded from the final model.

### Data analysis

The analytical sample was limited to respondents who were interviewed at enrollment and follow-up, were 16–49 years of age, and were using FP as a result of their visit to a CP or PPMV and were not currently pregnant (n = 491). Respondents who were pregnant at the time of the follow-up interview (n = 17), who were 50 years or older (n = 5), who did not receive a method from a CP/PPMV or from CP/PPMVs’ referral (n = 3), and those who did not answer the motivation question (n = 1) were excluded. Descriptive statistics were calculated for respondent characteristics, FP use, changes in motivation to avoid pregnancy between enrollment and follow-up interviews, and experience of side effects at follow-up.

Multivariate models that accounted for the longitudinal nature of the data were conducted but the likelihood ratio test showed that most of variance in the random intercept was accounted for by the covariates. Univariate and multivariate logistic regression models were used to assess the association between motivation to prevent pregnancy and contraceptive continuation. The logistic regression models were also run with women regardless of pregnancy status at follow-up, and all women regardless of age. The results from these sensitivity analyses were not statistically different from the final model. The analyses were conducted in STATA.SE, Version 16.

## Results

Table 1 presents the demographic profile of women who received FP services from CPs and PPMVs at the enrollment interview. Most (86%) women were between 25 and 49 years of age: 47% were 25–34 and 39% were 35–49. Many had not obtained a secondary education or higher (76%) and were employed at the time of the enrollment interview (72%). The majority of women were married (95%) and had two or more living children (86%). A little over half (56%) had previously used FP in the past. Half of the women were from Kaduna and half from Lagos. Seventy-seven percent of women received their method from a PPMV and 23% from a CP (data not shown).

**Table 1:** Respondent characteristics at enrollment, and experience of side effects and contraceptive continuation and at follow-up (n = 491)

	#	%
Age		
16–24	68	13.9
25–34	231	47.0
35–49	192	39.1
Education		
Did not complete secondary education	367	74.7
Completed secondary education or higher	124	25.3
Currently employed		
Yes	355	72.3
No	136	27.7
Marital status		
Not currently married	27	5.5
Currently married	464	94.5
Number of living children		
None	17	3.5
1	53	10.8
2	92	18.7
3	138	28.1
4 +	191	38.9
Has used FP in the past		
Yes	273	55.6
No	218	44.4
Motivation to avoid pregnancy		
Remained highly motivated	324	66.0
Increase in motivation	73	14.9
Decrease in motivation	72	14.7
Remained motivated/low motivation	22	4.5
State of residence		
Kaduna	49.7	49.7
Lagos	50.3	50.3
Experienced of side effects		
Yes	163	33.2
No	307	62.5
No response	21	4.3
Continued to use a FP method		
Yes	438	89.2
No	53	10.8

Two-thirds of women were highly motivated to avoid pregnancy at both interviews. Approximately 15% became more motivated at follow-up and 15% became less motivated. Among the women who became more motivated (n = 74), 64% increased from motivated to highly motivated, 27% went from not motivated to highly motivated and 8% went from not motivated to motivated (data not shown). Among the women who became less motivated (n = 73), 73% went from highly motivated to motivated, 20% went from highly motivated from not motivated and 8% from motivated to not motivated (data not shown). At follow-up, one-third (33%) reported experiencing side effects and approximately 89% of women continued to use contraception (82% reported using the same method and 7% reported switching to another method, data not shown). Of the 54 women who reported discontinuing FP, 21 (39%) said the main reasons was due to side effects, 15 (28%) expressed a desire to become pregnant, 4 reported the method was inconvenient (7%), and the remaining 13 reported one of a variety of other reasons (data not shown).

**Fig. 1:**
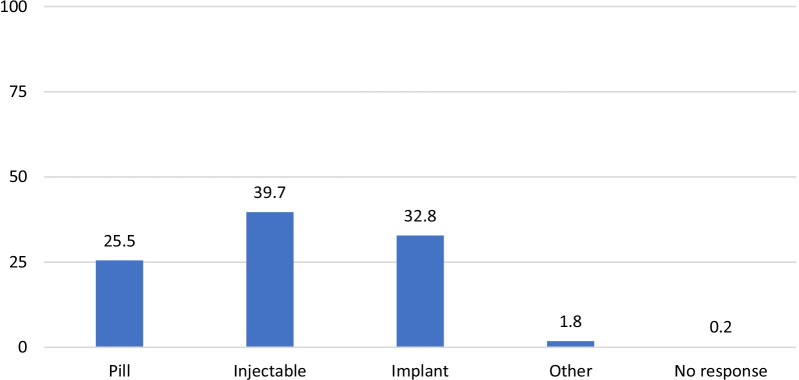
Distribution of current method use as reported at enrollment (N = 491)

Figure 1 shows the distribution of current method use as reported at the enrollment interview. Two-fifths (40%) of women were using the injectable, 33% were using an implant and 24% were using the pill. Two percent reported using another method such as condoms, cycle beads, and or an IUD (as a result of a referral).

**Fig. 2:**
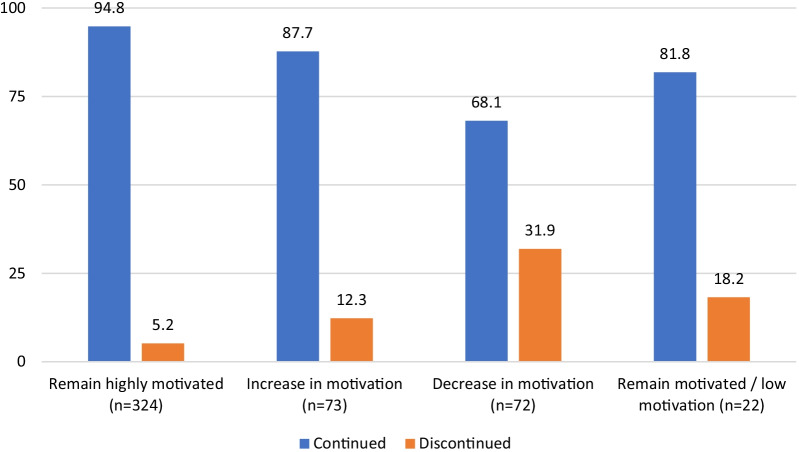
Distribution of continuers and discontinuers at follow-up by their fertility intentions as reported at enrollment (N = 491)

Figure 2 shows the distribution of continuers and discontinuers at follow-up by their motivation to avoid pregnancy. Among women who remained highly motivated at both interviews, 95% continued using contraception approximately 9 months later compared to 88% of women who became more motivated at the time of the follow-up. Only 68% of women who became less motivated continued using their contraceptive method. About 82% of those who remained motivated or remained unmotivated continued using contraception.

Unadjusted and adjusted odds ratios of contraceptive continuation are presented in Table 2. Women who remained highly motivated to prevent pregnancy were 2.5 times more likely to continue using contraception compared to those who reported an increase in motivation (OR = 2.5; 95% CI 1.1–6.0). Women who reported a decrease in motivation to prevent pregnancy were 70% less likely to be using contraception compared those who reported an increase (OR = 0.30; 95% CI 0.1–0.7). When accounting for covariates in the multivariate model, women who remained highly motivated were significantly more likely to be using contraception compared to women who became more motivated (AOR 2.5; 95% CI 1.0–6.0). Women who became less motivated were significant less likely to be using contraception compared to those who became more motivated, but the significance decreased slightly from the univariate model (AOR 0.4; 95% CI 0.1–0.9). Women who did not experience side effects at follow-up were 2 times more likely to be using contraception compared to women who did in the univariate model (OR 2.1; 95% CI 1.2–4.0) and multivariate model (AOR 2.2; 95% CI 1.1–4.3).

**Table 2:** Univariate and multivariate logistic regression models of fertility intentions on contraceptive continuation among women who received FP services from CPs and PPMVs (n = 491)

	Univariate Model		Multivariate Model	
	OR	95% CI	AOR	95% CI
Motivation to prevent pregnancy at both interviews				
Remained highly motivated	2.54*	1.08–5.95	2.50*	1.03–6.01
Increase in motivation	Ref	–	Ref	–
Decrease in motivation	0.30**	0.13–0.71	0.36*	0.14–0.87
Remained motivated/low motivation	0.63	0.17–2.30	0.64	0.17–2.50
Age				
16–24	Ref	–	Ref	–
25–34	0.95	0.39–2.29	0.76	0.26–2.26
35–49	0.93	0.38–2.31	0.68	0.20–2.30
Education				
Did not complete secondary education	Ref	–	Ref	–
Completed secondary education or higher	1.74	0.82–3.68	1.49	0.66–3.37
Currently employed				
Yes	1.03	0.55–1.95	1.39	0.63–3.11
No	Ref	–	Ref	–
Marital status				
Not currently married	Ref	–	Ref	–
Currently married	1.47	0.49–4.44	0.69	0.14–3.53
Number of living children				
None	0.20**	0.06–0.66	0.35	0.06–2.02
1	0.56	0.22–1.45	0.68	0.21–2.13
2	0.57	0.25–1.27	0.87	0.35–2.18
3	0.75	0.35–1.62	1.16	0.50–2.70
4 +	Ref	–	Ref	–
Have ever used FP				
Yes	1.13	0.64–2.01	1.10	0.58–2.09
No	Ref	–	Ref	–
Experience of side effects*				
Yes	Ref	–	Ref	–
No	2.13*	1.15–3.96	2.17*	1.10–4.29
Did not respond	0.27**	0.10–0.71	0.44	0.14–1.39
State of residence				
Lagos	Ref	–	Ref	–
Kaduna	1.58	0.88–2.83	0.92	0.42–2.04

*p-value ≥ 0.05; ** p-value ≥ 0.01

## Discussion

Results from this analysis suggest that women’s motivations to prevent pregnancy are associated with contraceptive continuation. Specifically, women who remained highly motivated were more likely to continue using contraception compared to those who became more motivated. These results contribute to the growing body of work that looks at women’s pregnancy motivations and are consistent with previous studies that found associations between motivation to avoid pregnancy and contraceptive use and continuation [18–20]. This is one of the first analyses, however, to look at how changes in motivation can also influence contraceptive continuation.

Many women in this sample remained highly motivated to prevent pregnancy. For about 30%, however, their motivations either decreased or increased within a year. As highlighted in previous studies [17, 18, 35], our results also suggest that it is important for healthcare providers to continually ask their clients about their pregnancy motivations due to the association with continuation. Pregnancy motivations, like fertility intentions, have also been documented to change not only over woman’s life course, but also within a short period time due to unexpected life changes like unemployment or illness [17, 35] By continually checking in with women, providers can assist their clients to choose the most appropriate method or switch to a different method if, and when, their life and motivations change. For women who become more or less motivated, discussing the possibility of switching to a longer/shorting acting method may be appropriate, especially if the woman is not happy or satisfied with her current method. Similarly, women who remain motivated but are unsatisfied with their method (because of side effects or the method is inconvenient) should have the option to switch to a method that meets their needs. This will be especially important for “new” FP providers such as CPs and PPMVs as the Federal Ministry of Health continues to explore including them in their FP task sharing policy. More broadly, all reproductive healthcare providers and FP programs could also explore adding questions around pregnancy motivations to their FP counseling protocols, given the additional nuance this question can provide.

Both motivation to avoid pregnancy and experience of side effects were significantly associated with continuation in the multivariate model. When asked for the main reason why women discontinued, the two main reasons cited were side effects/health concerns and desire to become pregnant, similar to the reasons documented nationally [23]. This suggests that there may be more than one factor influencing women’s decision to continue using contraception. Discussing women’s pregnancy motivations, in addition to their fertility intentions and side effects, are important for comprehensive FP counseling. This is especially for women who are motivated to avoid pregnancy but unhappy with their method due to side effects, or other reasons, and could switch to an alternative method.

Another important finding from this study is that 89% of women in need who received a FP method as a result of a visit from CPs and PPMVs continued to use a FP about 9 months later. Previous studies have documented proximity and flexible operating hours as reasons why women prefer PPMVs for FP and other primary health care services [37, 38]. Results from our study further suggest that when properly trained, CPs and PPMVs may also facilitate women’s continued use FP. These results underscore the importance of training CPs and PPMVs to facilitate conversations around pregnancy motivations, fertility intentions, and method-related characteristics with their clients.

### Limitations

Data for this analysis came from a specific population of women who sought FP services from the CPs and PPMVs and therefore the results are not representative of the broader population of Nigerian women of reproductive age. These women were also motivated to avoid pregnancy because they voluntarily sought FP services from a CP or PPMV and had little variation in terms of motivation and respondent characteristics such as marital status. Our results, however, are consistent with other studies that have found an association between motivations to avoid pregnancy, and contraceptive use and continuation. The results from this study contribute to the literature by documenting how motivations to avoid pregnancy can change over a short period of time and how those changes are associated with contraceptive continuation. This study also provides a unique perspective of women who seek services from private sector pharmacies and drug shops (PPMVs). Additional research that builds off these results and explores changes in motivation and contraceptive continuation among a broader population should be explored.

Another potential limitation is that respondents had to own a phone to be eligible to participate, which may have introduced a form of selection bias although phone ownership by women is relatively high (83%) in Nigeria. This criterion was included to reduce the number of respondents lost to follow-up since we used phone interviews. Owning a phone was used as the eligibility criteria over access to a phone to reduce any potential of disclosing respondents’ contraceptive use to others when the research assistants called for the interview.

## Conclusion

Many women who seek FP services from private sector CPs and PPMVs in Nigeria are highly motivated to avoid pregnancy. For some of these women, however, their pregnancy motivations change within a short period of time. As motivation to avoid pregnancy was significantly associated with continuation among this population, supporting CPs and PPMVs to provide comprehensive FP that facilitates a dialogue with their clients about their pregnancy motivations, will be essential for a successfully task sharing policy.

## Data Availability

The dataset analyzed during the current study will be available on the Population Council’s Dataverse, https://dataverse.harvard.edu/dataverse/popcouncil in January 2022. They will also be available from the corresponding author on reasonable request.

## Annex 1

Comparison of characteristics between women who were lost-to follow up and women interviewed at both time points

**Table:**

	Lost to follow up (n = 77)	Not lost to follow up (n = 517)
Age		
16–24	11.3	88.7
25–34	14.9	85.1
35–49	11.1	88.9
Education		
Did not complete secondary education	12.8	87.2
Completed secondary education or higher	13.4	86.6
Currently employed		
Yes	12.2	87.8
No	13.3	86.7
Marital status		
Not currently married	21.4	78.6
Currently married	12.3	87.7
Number of living children		
None	13.6	86.4
1	10.0	90.0
2	15.8	84.2
3	11.5	88.6
4 +	13.4	86.6
Has used FP in the past		
Yes	13.0	87.0
No	13.0	87.0
State of residence		
Kaduna	11.2	88.8
Lagos	14.7	85.3

## Abbreviations

CP: Community pharmacist
FP: Family planning
PPMV: Patent and proprietary medicine vendor
